# A semi-continuum model of saturation overshoot in one dimensional unsaturated porous media flow

**DOI:** 10.1038/s41598-019-44831-x

**Published:** 2019-06-10

**Authors:** Jakub Kmec, Tomáš Fürst, Rostislav Vodák, Miloslav Šír

**Affiliations:** 1 Palacky University in Olomouc, Joint laboratory of optics, Faculty of Science, Olomouc, 771 46 Czech Republic; 2 Palacky University in Olomouc, Dpt. Mathematical analysis and applications of mathematics, Faculty of Science, Olomouc, 771 46 Czech Republic; 3 University of South Bohemia, Institute of Aquaculture and Protection of Waters, Faculty of Fisheries and Protection of Waters, České Budějovice, 370 05 Czech Republic

**Keywords:** Hydrology, Mathematics and computing

## Abstract

A semi-continuum model for fluid flow in saturated-unsaturated porous medium in one spatial dimension is presented. The model is based on well-established physics, measurable parameters and material characteristics. The porous material is characterized by porosity, intrinsic permeability, main wetting and draining branches of the retention curve, and the saturation dependence of the relative permeability. The fluid is characterized by its density and dynamic viscosity. The only physics involved is the mass balance of fluid in porous media together with the Darcy-Buckingham Law for fluid flow in unsaturated porous media. The model is a cellular automaton based on the Macro Modified Invasion Percolation concept of dividing the porous medium into blocks which are not infinitesimal and are assumed to retain the characteristics of a porous medium. The cellular automaton repeats three successive rules: saturation update in each block, pressure update in each block, and flux update between neighboring blocks. The model tracks the evolution of the relative saturation, the fluid capillary pressure, and the fluid flux. The model is shown to reproduce qualitatively and quantitatively all features of one dimensional saturation overshoot behavior reported in the literature.

## Introduction

Traditionally, porous media flow is described in the framework of continuum mechanics^1^. The continuum mechanics based models lead to partial differential equations which are mathematical formulations of the balance of mass, momentum, and energy. The most used model connected to porous media flow is the so called Richards’ Equation (RE)^2^.

However, there are several persistent and important flow regimes in unsaturated homogeneous porous media (UHPM) which are not easily captured by continuum-mechanics based modeling^3^. All of the regimes share the following setting: The porous medium is homogeneous, i.e. it does not contain any macropores, cracks, or preferential channels. The porous medium is unsaturated, i.e. some of the pores are filled with wetting fluid and some with gas. Gravity and capillary forces both play important roles and neither can be neglected.

**Figure 1:**
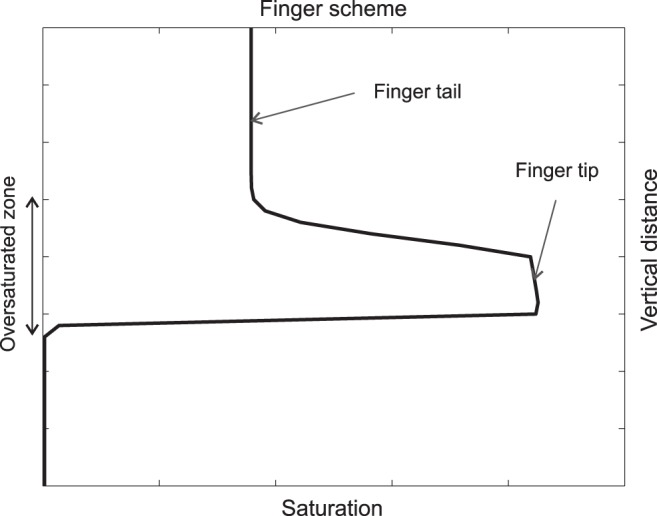
A schematic representation of saturation overshoot in a single finger.

The so-called finger flow, or fingering effect, rates among the most intriguing of such regimes. The regime can be reached e.g. as follows: Let us start with a sample of initially dry homogeneous porous medium. Wetting fluid is supplied to one point at the upper boundary of the sample at a small constant rate. Under certain conditions (see below), a single (macroscopic) finger forms and proceeds downwards at almost constant speed (see Fig. 1).

The proceeding finger leaves an under-saturated trace. Subsequent infiltration follows the path wetted by the first finger. The key feature of this regime is the non-monotonicity of the saturation: At certain points (through which the finger tip passes) saturation is a non-monotone function of time, first it increases abruptly as the over-saturated fingertip arrives, and then it gradually decreases again as the under-saturated fingertail passes. This effect is called the saturation overshoot.

Saturation overshoot in gravity-driven fingers was experimentally observed by Glass *et al*.^4^, Liu *et al*.^5^ and Selker *et al*.^6^. Comprehensive experimental work was done by DiCarlo *et al*.^7–9^. The main experimental results can be summarized as follows:

- Fingering accompanied by saturation overshoot is observed in wide range of homogeneous porous media types–soils, sands, and artificial glass beads, uniformly or randomly packed. It is observed for wide range of flow rates, and for wide range of initial saturation levels.
- The overshoot effect is observed in three-dimensional, two dimensional (Hele-Shaw cells) and one-dimensional (narrow tubes) settings.
- The finger behavior depends on the initial saturation of the matrix. With increasing initial saturation, the fingers first become more narrow and faster, then they widen and slow down and the saturation overshoot decreases. The overshoot phenomenon disappears when the initial saturation exceeds a certain limit. For initial saturation close to the residual moisture limit, the fingering regime disappears and diffusion-like front forms with no overshoot.
- The saturation overshoot magnitude (i.e. the difference between the saturation in the tip and the saturation in the tail) increases with increasing flow rate up to a certain value, beyond which it decreases until it disappears completely. However, there is also a lower limit–saturation overshoot is not observed for very small flow rates.
- Saturation overshoot depends on the shape of the porous matrix grains. The effect is less pronounced for spherical sand grains than for sands with more angular (irregular) shapes. Materials with very similar macroscopic continuum properties exhibit different saturation overshoot patterns.
- The finger-tip is not always fully saturated, its saturation depends on the flow rate and on initial saturation of the medium.
- Capillary pressure overshoot is consistent with the saturation overshoot, and the pressure-saturation relation does not seem to depend on the finger velocity, although other sources^10^ report some evidence for velocity dependent behavior.
- The overshoot behavior depends on the ratio of capillary and gravitational forces captured by the so-called Bond number^11^.
  There have been many attempts to capture gravity-driven fingers and especially the saturation overshoot by continuum mechanics based models, e.g. adding new terms into the RE^12–16^. The equation used for UHPM flow is the Richards’ Equation (RE)^2^ which can be stated in the following form:
  1 $${\partial }_{t}S(t,x)={\rm{div}}\,[K(S(t,x))\nabla h(S(t,x))+(0,0,K(S(t,x)))].$$
  Here *S*(*t*, *x*) is the unknown field of relative moisture content (the saturation of the wetting phase, dimensionless, between 0 and 1), *K* stands for the hydraulic conductivity which is assumed to be non-negative and non-decreasing function of the saturation. The function $h:\mathrm{[0},1]\to {\mathbb{R}}$ represents the so called retention curve–i.e. the dependence of the capillary height on the moisture content. The retention curve is assumed to be non-decreasing and it is known to exhibit substantial hysteresis. The capillary pressure *P*_*w*_(*S*) is related to the retention curve by the simple rescaling relation
  $${P}_{w}(S)=\rho gh(S),$$
  where *ρ* is the density of fluid and *g* is gravity.
  There have been many attempts to add new terms into the RE in order to describe saturation oveshoot^12–16^ or to use the RE to describe at least some of the features of finger flow^6^.
  Let us now turn to a completely different possibility of UHPM flow modeling, which we call discrete modeling. The interest in discrete dynamical systems goes back to the work of Ulam^17^ and von Neumann^18^. The field of discrete models, often called cellular automata, was usually considered a part of statistical physics, and had almost no overlap with porous media flow modeling. In the 1970s, a new branch of discrete dynamical systems science emerged, which became known as percolation theory. The most important early contributions can be traced back to Kirkpatrick^19^ and Stauffer^20^. It was soon recognized that percolation theory can be used to model immiscible fluid flow in porous media. The theory was used to predict the fractal structure of the percolating clusters of fluids, capture the critical behavior of pressure and hydraulic conductivity dependence on saturation near the percolation threshold, and point out the universality of the various scaling relations^21^. Lenormand *et al*. conducted an extensive research in this direction, both experimental and theoretical, which clarified the mechanisms by which individual pores are filled and drained by wetting and non-wetting fluids^22–25^, see also the work of Blunt and Scher^26^ for important contributions to the understanding of collective pore filling mechanisms. Based on these seminal papers, there has been an explosion of theoretical and experimental results which used percolation theory to capture the flow of immiscible fluids in two or there dimensional porous media at the pore level, under various combinations of viscous and capillary forces and the force of gravity. The porous medium is usually modeled as two or three dimensional regular or random network of pores and/or throats, of various types of cross-sectional shapes and various types of connectivity. Usually two (sometimes three) immiscible fluids are allowed to move inside this network. Pores and throats are usually filled with one fluid, or the other, intermediate levels of saturation inside a single pore are seldom considered. These models are typically called Invasion Percolation (IP) models or, if they also include an external force field, Modified Invasion Percolation (MIP).
  It is natural to try to combine the advantages of both the continuum-based and discrete approaches. Let us call these model *semi-continuum models* borrowing the term from the article of DiCarlo *et al*.^27^. A semi-continuum model was reported by Glass *et al*.^28^ following a series of papers^29–32^. Let us note that the authors themselves call the approach “mechanistic modeling”, or Macro Modified Invasion Percolation (MMIP), they never use the term “semi-continuum model”. In their MMIP model, the porous medium is represented as a regular grid of rectangular sites (called blocks) in two or three dimensions, with certain specified type of connectivity. Each block is a small part of the original porous medium (hence our “semi-continuum” label) completely described by two numbers:
- *P*_*w*_ is the pressure needed for the invading phase to fully percolate the block (that means to form a connected network of filled pores throughout the block so that the block becomes conductive for that phase).
- *P*_*d*_ is the pressure needed for the defending (retreating) phase to reinvade the block.

The pressure can be related to a radius of curvature by means of the formula

2 $${P}_{i}=-\frac{2\sigma \,\cos \,\varphi }{{R}_{i}},$$

where *i* stands either for *w* or *d*, *σ* is the surface tension between the phases and *ϕ* is the contact angle. Thus, each block can be assigned a critical wetting radius *R*_*w*_ and a critical draining radius *R*_*d*_.

The MMIP conceptualization has the great advantage (over IP nad MIP models) of not having to worry about the particular geometry of the pores and throats. It is important to note that the radii *R*_*i*_ in Eq. (2) do not represent any dimension of any actual (real) pore in the block–they represent the capillary pressures given in units of capillary radii. Each block is either full (percolated by the invading fluid) or empty (not percolated). The model is binary in nature as other IP and MIP models, it does not capture saturation as a continuous variable. The percolation rules for invasion and withdrawal are based on local pressure considerations and global gradient of the force of gravity. The process of facilitation (preferential filling of pores which are surrounded by already full pores) is included if the invading phase is wetting. The model is reasonably simple, it does not introduce any free parameters (not related to measurable properties of the porous medium) and it is able to capture three very different flow regimes by means of a unified approach.

We want to present a slightly different semi-continuum model in one spatial dimension. This model reproduces qualitatively and quantitatively all of the features of saturation overshoot behavior reported by DiCarlo^33^. The model is based on well-established physics only. In the next section, the physics of the model is presented and the resulting cellular automaton is fully described. In the Results section, the ability of the model to reproduce experimental observations is presented. Saturation and capillary pressure overshoot behavior is reproduced, both qualitatively and quantitatively. The dependence of the saturation overshoot on influx and initial saturation is reproduced, too.

## Methods

Let us suppose a long narrow vertical tube filled with homogeneous porous medium, e.g. 20/30 sand as in the experiments of DiCarlo^33^. The tube has a cross-section of *A* [m^2^], the height of the tube is *L* [m]. Lets subdivide the tube into small blocks of height *dx* and cross sectional area *A*. These blocks represent “pieces” of the original porous medium and they are not to be considered infinitesimal. For a given time, the key physical quantities (see below) are considered constant within a single block. This is a direct reference to the semi-continuum approach of Glass and Yarrington^28^. The tube now consists of slices [*ndx*,(*n* + 1)*dx*] with *n* = 0,1, … *N*. The key quantities that we want to track are

- Saturation *S*_*i*_ [−] in each block.
- Capillary pressure *P*_*i*_ [Pa] in each block i.e. the average pressure difference across the menisci (wetting fluid–gas interfaces) in the block. For simplicity, air pressure is set to zero everywhere.
- Fluxes *q*_*i*,*j*_ [m/s] between the blocks *i* and *j*. Real fluxes in [m^3^/s] can be recovered as *Aq*_*i*,*j*_. Naturally, only fluxes between neighboring blocks are non-zero in this setting.

Gravity is directed downward along the long axis of the tube, which is called the *x*− axis here. A constant (in time) influx *q*_0_ [m/s] at the top boundary (*x* = 0) is assumed. Zero discharge *q*_*L*_ = 0 (i.e. zero flux) at the bottom boundary (*x* = *L*) is assumed.

### Saturation update

Naturally, a mass balance has to hold. Here, we use the form

$$\theta {\partial }_{t}S(t,x)+{\partial }_{x}q(t,x)=0,$$

where *θ* [−] stands for the porosity of the material. This can be understood in the semi-continuum setting as a simple discrete scheme

3 $$\frac{\theta }{dt}[{S}_{i}(t)-{S}_{i}(t-dt)]=\frac{1}{dx}[{q}_{i-1,i}(t-dt)-{q}_{i,i+1}(t-dt)],$$

where *dx* and *dt* are discretization parameters. The specific form of the fluxes *q*_*ij*_ are given by (7). So, knowing the fluxes at time *t*−*dt*, one can update the saturation in each block in this straight forward way.

### Pressure update

Next, the pressure in each block has to be updated, because the fluxes are governed by pressure gradients. Hysteresis of the retention curve has to be addressed in this step because it is known^34^ that, in the case of fingering, the (over-saturated) finger tip is in the imbibition mode while the under-saturated finger tail is in the draining mode. Consequently, no reasonable model of saturation overshoot can ignore hysteresis. Let us use the following very simple approach. Suppose that the material has a well-defined main wetting branch of the retention curve denoted by

4 $${P}_{i}(t)={F}_{W}({S}_{i}(t\mathrm{)).}$$

This means that if a block starts at zero saturation and becomes more and more wet, the capillary pressure in the block will be dependent on its saturation through Eq. (4). Analogously, there is the main draining branch of the retention curve

5 $${P}_{i}(t)={F}_{D}({S}_{i}(t\mathrm{)).}$$

**Figure 2:**
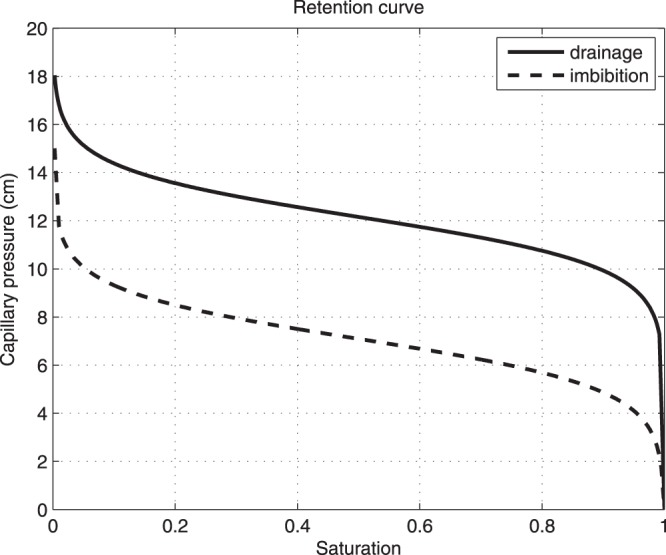
The retention curve used in our model. The curve roughly matches 20/30 sand used in the article of DiCarlo^33^. Capillary pressure given as pressure head in cm of water column.

For any block that starts at unit saturation and becomes less and less wet, the capillary pressure will be given by (5). A typical retention curve of a 20/30 sand is shown in Fig. 2. The wetting and draining branch are both modeled by the logistic functions here with small corrections at the *S* = 0 and *S* = 1 ends.

If a block first undergoes imbibition but then switches to draining, it moves in the saturation-pressure diagram (Fig. 2) between the two main branches. In experiments, significant changes in pressure are observed with almost no change in saturation^33^. We adopt the simplest modeling approach and describe this process by a relation

6 $$\frac{dP}{dS}={K}_{PS}$$

with some large constant *K*_*PS*_. It means that between the main branches, saturation changes with pressure very rapidly, following a line with a very large slope *K*_*PS*_. One may think of the saturation jumping from the wetting to the draining branch discontinuously (i.e. vertically) but this would cause unnecessary numerical issues in the model, hence this approximation. We believe there is a simple explanation for this rapid behavior: Capillary pressure originates as an average of the pressure drops across the fluid–gas menisci. As a block switches between imbibition and draining, the shape of the menisci changes (they become more pronounced with smaller radii) which causes almost no measurable change in volume but a dramatic increase in the pressure drop across the menisci. Relation (6) holds anywhere between the main wetting and the main draining branches. Once a block in the draining mode reaches the main draining branch, it sticks to it and continues along it. Analogously, once a block in the imbibition mode reaches the main wetting branch, it sticks to it. In this way, we update the pressures in all the blocks using the current (already updated) levels of saturation.

### Flux update

At last, we have to update the fluxes. This is where we slightly depart from the usual Richards’ Equation formulation (1). It is understood that the advancing of the finger tip is where the classical theory fails. The overshoot can be understood as fluid piling up behind the fingertip because the dry medium ahead of the fingertip has insufficient hydraulic conductance thus the flux across the fingertip cannot keep up with the flux in the tail of the finger. Thus, fluid piles up behind the fingertip which increases the hydraulic gradient across the finger tip until the large gradient “matches” the low conductivity and the flux across the fingertip equals the flux in the tail. Then, a stable finger with an oversaturated tip proceeds with constant velocity downwards.

The original Darcy-Buckingham Law for unsaturated porous media flow^1^ states:

$$q=\frac{\kappa }{\mu }k(S)(\rho g-\nabla P),$$

where *κ* [m^2^] is the intrinsic permeability of the medium, *μ* [Pas] is the dynamic viscosity of the fluid, *ρg* is the gradient of the gravitational potential, and *P* [Pa] is the capillary pressure. Here *k*(*S*) [−] stands for the relative permeability which is very sensitive to *S* and is usually modeled by a power law relation

$$k(S)={S}^{m}$$

with *m* usually around 3 or 4 (some sources report *m* as high as 10!^16^ but since saturation may vary over three orders of magnitude this would result in relative permeability varying over 30 orders of magnitude, which may not be reasonable).

In view of the two preceding paragraphs, it is crucial how we model the conductance at the finger tip, where ∇*P* is large, and *S* changes abruptly from small values in front of the fingertip to large values inside the fingertip. We propose the following discrete implementation of the Darcy Law:

7 $${q}_{i,j}(t)=\{\begin{array}{ll}\frac{\kappa }{\mu }\sqrt{k({S}_{i}(t))k({S}_{j}(t))}(\rho g-\frac{{P}_{j}(t)-{P}_{i}(t)}{dx}) & \,{\rm{for}}\,j=i+1\\ 0 & \,{\rm{otherwise}}\,\end{array}$$

Thus, for the relative permeability at the finger tip edge, we simply take the *geometric* mean of the permeability of the respective blocks. The geometric mean $\sqrt{ab}$ has the desirable property of being small if one of the numbers *a* and *b* is small. It is also possible to use the *harmonic* mean with similar results. The more common (arithmetic) mean does not behave in this way. Notice that if the saturation vanishes in a block, the flux to/from the neighboring blocks becomes zero. Therefore, the initial saturation in all blocks has to be set to a nonzero value.

Notice that in the limit *dt* → 0, *dx* → 0, the numerical scheme (with geometric or arithmetic mean) converges back to RE which is known^35^ to be incapable of producing any overshoot. Thus, the issue of the limit of this semi-continuum model is an important one and it is left to the Discussion section.

### Model description

The resulting cellular automaton works as follows:

1. The size of the blocks *dx* is chosen and an appropriate time step *dt* is set (see Discussion). Initial saturation *S*_*i*_ is prescribed in each block (the same in all the blocks), the corresponding capillary pressure is computed, and all fluxes are initially set to zero.
2. The top boundary condition is set: the flux into the topmost block is set and fixed to *q*_0_. The bottom boundary condition is set: the flux out of the bottom block is set and fixed to zero.
3. Using the current value of the fluxes *q*_*i*,*j*_, saturation *S*_*i*_ in each block is updated according to Eq. (3).
4. Pressure *P*_*i*_ in each block is updated according to Eqs (4)–(6), keeping track whether the block is in the imbibition or draining mode.
5. Fluxes *q*_*i*,*j*_ between neighboring blocks are updated according to (7), keeping the boundary fluxes fixed by step 2.
6. Time is updated to *t* + *dt* and the process goes back to step 3.

## Results

In this section, we reproduce the experimental results of DiCarlo^33^. The experiments were performed by DiCarlo *et al*. with water infiltrating a narrow tube filled with 20/30 sand. The diameter of the test tube was chosen by DiCarlo so that the saturation in the column was uniform transverse to the flow direction at all times. There was no observable preferential flow in the columns, as the diameter was less than the finger diameter. The tight packing of the sand ensured there were no observable effects of preferential flow along the tube walls. A tube with the inner diameter of 1.27 cm was used. To keep close to the experimental setting (see the Materials and Methods section in the article of DiCarlo^33^), the following parameters are used for the simulation:

- porosity of the material *θ* = 0.35 [−],
- intrinsic permeability of the material *κ* = 1 × 10^−10^ m^2^,
- dynamic viscosity of water *μ* = 9 × 10^−4^ Pas,
- density of water *ρ* = 1000 kg/m ^3^,
- acceleration due to gravity *g* = 9.81 m/s^2^,
- cross sectional area of the tube *A* = 10^−4^ m^2^ (this will not be needed in the model because it is one dimensional),
- length of the tube *L* = 40 cm (the tube will be divided into 40 blocks of height 1 cm).

The choice of *dx* is inspired by Glass and Yarrington^28^ and reflects the requirement for the blocks to be large enough to retain the characteristics of the original porous medium. At the begining, all blocks are assumed to be in imbibition mode. The saturation dependence of the relative permeability is modeled by *k*(*S*) = *S*^3^. The retention curve in Fig. 2 is used with *K*_*PS*_ = 10^5^ Pa.

### Saturation overshoot in initially dry medium and its dependence on the influx

**Figure 3:**
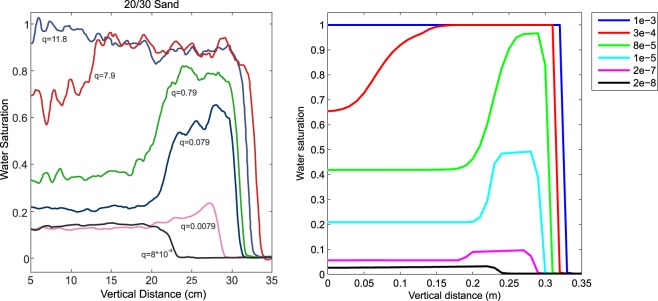
Comparison of the saturation profiles for various influx rates. Left panel: Experimental data, infiltration into a narrow tube of initially dry 20/30 sand, reprinted from the article of DiCarlo^33^ (Fig. 2), fluxes are reported in cm/min into a test tube of inner diameter of 1.27 cm. Republished with permission of Soil Science Society of America, from DiCarlo, D. A. Can continuum extensions to multiphase flow models describe preferential flow? Vadose Zone J. 9(2), 268–277 (2010). doi 10.2136/vzj2009.0099; permission conveyed through Copyright Clearance Center, Inc. Right panel: One dimensional simulation of the same situation by the semi-continuum model, initial saturation *S*_*i*_ = 0.003, fluxes are reported in m/s into a system of cross-sectional area of 1 cm^2^.

First, we want to demonstrate the ability of the semi-continuum model to capture saturation overshoot in infiltration into initially dry homogeneous porous medium, and the dependence of the overshoot on the influx. The dependence of the oversaturation profile on influx is rather complicated: For a very small influx, there is no visible saturation overshoot. With increasing influx, a distinct overshoot pattern appears. The magnitude of the overshoot and the length of the oversaturated zone increases with increasing influx. If the influx is too large, the fingertip becomes almost completely saturated and the length of the oversaturated zone grows all the way to the upper boundary of the sample. Thus, the overshoot disappears for fluxes which are too large. This rather complicated dependence is well replicated by the semi-continuum model, see the comparison of the experimental data by DiCarlo^33^ in the left panel of Fig. 3 and our numerical simulation in the right panel of the figure. For technical reasons, we cannot prescribe initial saturation to be zero (this would yield zero hydraulic conductivity) so we chose *S*_*i*_ = 0.003 everywhere. Since we report the results in SI units and assume the cross-sectional area of the tube to be *A* = 1 cm^2^, the conversion factor for the influx between the experiment and the simulation is roughly 60 × 100 × 1.27^2^ ≅ 10000. Thus, the experimental influx that was small enough not to cause saturation overshoot (*q*_0_ = 8 × 10^−4^ cm/min) corresponds roughly to *q*_0_ = 2 × 10^−8^ m/s in our simulation. Further, the experimental flux which causes complete flooding of the material (*q*_0_ = 11.8 cm/min) with no overshoot roughly matches the same situation in our simulation (*q*_0_ = 10^−3^ m/s). It can be observed that the model is able to reproduce both the dependencies, magnitude of the overshoot and the length of the oversaturated zone.

### The effect of initial saturation

**Figure 4:**
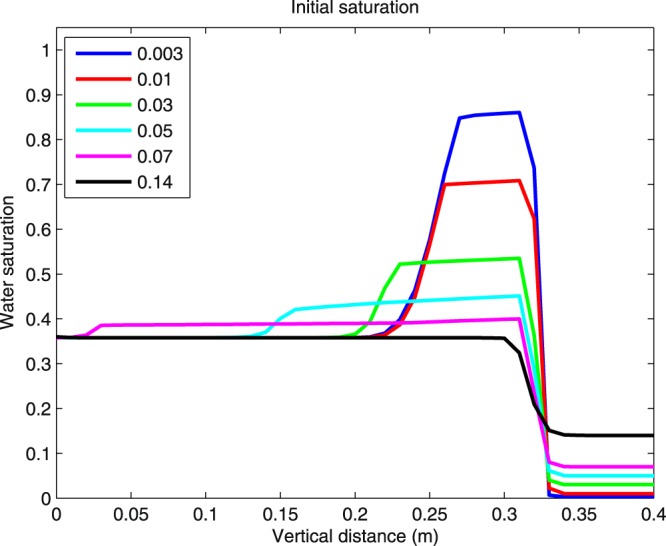
Comparison of saturation profiles for various values of initial saturation. Simulation with a constant influx of *q*_0_ = 5 × 10^−5^ m/s.

Let us now investigate the effect of initial saturation on the saturation overshoot. It is known^9^ that with increasing initial saturation, the overshoot is less and less pronounced and finally disappears completely, and diffusion-like behavior prevails. This seems to be captured well by the semi-continuum model, see Fig. 4. In the experiments reportec by DiCarlo^33^, the overshoot disappears completely for *S*_*i*_ = 0.14, for influx comparable to our simulation. This is in a quantitative agreement with our simulation (see Fig. 4). Let us further observe that in the simulation, the tail saturation remains independent of the initial saturation *S*_*i*_. This is in agreement with the experimental results of Fritz^10^ (see Figure 6.3 in this article).

### Capillary pressure overshoot

**Figure 5:**
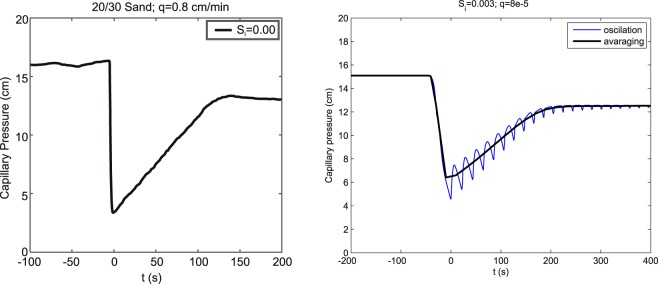
Evolution of the capillary pressure at a single point in the 13 th block from the top. Capillary pressure given as pressure head in cm of water column. The time is offset so that *t* = 0 corresponds to the arrival of the finger tip. Left panel: Experimental data reprinted from the article of DiCarlo^33^ (Fig. 5). Influx of *q*_0_ = 0.8 cm/min into initially dry 20/30 sand. Republished with permission of Soil Science Society of America, from DiCarlo, D. A. Can continuum extensions to multiphase flow models describe preferential flow? Vadose Zone J. 9(2), 268–277 (2010). doi 10.2136/vzj2009.0099; permission conveyed through Copyright Clearance Center, Inc. Right panel: Simulation by the semi-continuum model. The corresponding influx of *q*_0_ = 8 × 10^−5^ m/s into initially dry (*S*_*i*_ = 0.003) medium. The thin line shows the evolution of the capillary pressure at the point, the thick line shows the moving average over 20 seconds.

The capillary pressure overshoot is in agreement with saturation overshoot. If we observe the evolution of capillary pressure at a single point along the tube, and offset the time scale so that *t* = 0 corresponds to the arrival of the finger tip through this point, we obtain behavior depicted in Fig. 5.

Let us fix a point 12.5 cm from the upper boundary, i.e. a point in the 13 th block from the top. Let us offset the time so that *t* = 0 corresponds to the arrival of the fingertip at this point. We now track the evolution of the capillary pressure at this point. The time series of capillary pressure at this point exhibits visible oscillations after the finger tip passes. This is due to the build up of the saturation in the fingertip and then sudden burst into the block beneath. The solid line in Fig. 5 shows a 20 second moving average of the capillary pressure.

**Figure 6:**
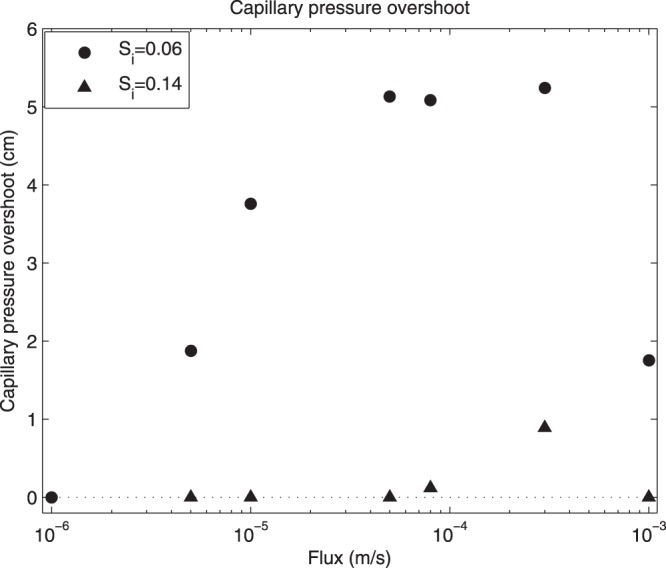
Capillary pressure overshoot as a function of the initial saturation and influx magnitude. The pressure overshoot is measured by the difference of the pressure in the finger tip and the pressure in the tail. Capillary pressure given as pressure head in cm of water column.

Although the pressure overshoot is consistent with the saturation overshoot, a more careful look reveals the following observation: DiCarlo^33^ reports that “When a larger initial water saturation of 0.06 was used, the [saturation] profiles are found to be monotonic at all fluxes, with no saturation overshoot”. However, a careful look at Fig. 8 of the original article^33^ (not reprinted here) shows that there is, in fact, a small capillary pressure overshoot for initial saturation 0.06 for fluxes higher than 10^−1^ cm/min. Even for initial saturation of 0.14 where no saturation overshoot was observed, the pressure overshoot is still clearly visible for high enough fluxes. This subtle behavior is well reproduced by our semi-continuum model (see Fig. 6).

The model reveals that, indeed, there is saturation overshoot accompanying *any* pressure overshoot but the saturation overshoot magnitude may be negligible (on the order of 10^−3^) and thus it cannot be observed in experiments. This explains the seemingly self-contradictory observation of DiCarlo.

### Finger build up

**Figure 7:**
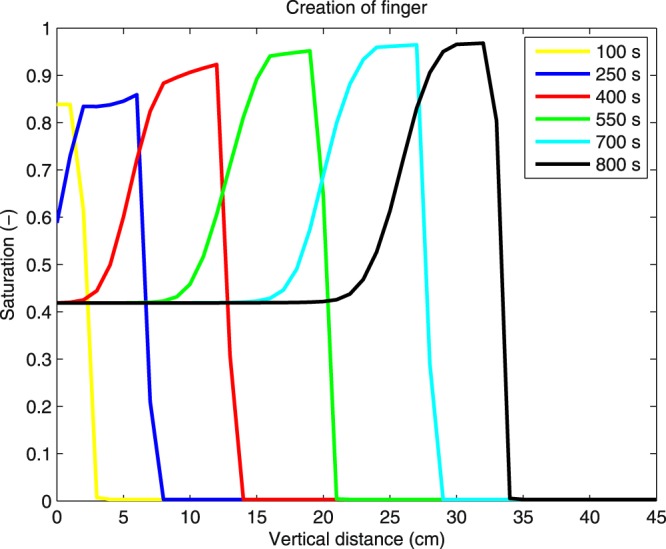
The process of finger build up. The profile of the finger at several successive time points.

To understand the formation of the finger well, it is convenient to plot the profile of the finger at various time points. Figure 7 shows the profile at several successive time points. The finger build up is a gradual process, both in space and in time. It takes several minutes before the saturation profile reaches its final (steady) shape. That is compatible with the experiments reported in the article of Fritz^10^, although the author expected, in contrast to his own experimental results, that the finger develops immediately at the inflow boundary and then flattens over time and depth.

**Figure 8:**
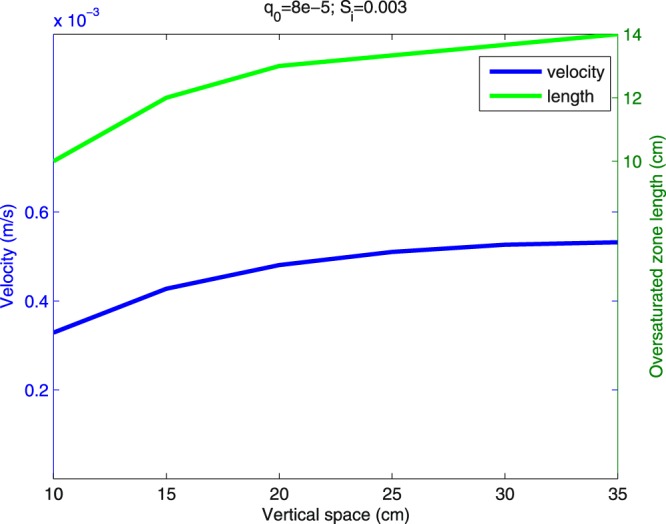
Evolution of the finger velocity and the length of the oversaturated zone. Compare with Fig. 6.7 in in the article of Fritz^10^.

Two more trends can be observed in the experiments reported in in the article of Fritz^10^ (Fig. 6.7): (1) the velocity of the finger tip gradually increases before reaching a steady state at a certain depth, (2) the length of the oversaturated zone of the finger increases with time (and thus also with the depth). Both these trends are correctly captured by the semi-continuum model (see Fig. 8).

## Discussion

First of all, it should be noted that the proposed model is not a numerical scheme for solving the Richards’ Equation (RE). RE in its original form (without new terms) was conclusively shown^35^ to be incapable of producing any overshoot for monotonic influx boundary conditions. If we perform the limit *dt* → 0, *dx* → 0 (keeping *dt*/*dx*^2^ constant, and *keeping permeability, porosity, and the retention curve of all the blocks constant*), the model converges to the RE and, accordingly, the overshoot behavior disappears. This convergence is theoretically clear from the statement of the model and has been tested numerically. Thus, this model is interpreted as a cellular automaton (or Macro Modified Invasion Percolation model) with continuous levels of saturation. We conjecture that the continuous limit of the cellular automaton must be approached in a different way.

In performing the limit *dt* → 0, *dx* → 0, one cannot assume that the distribution of the characteristics of the blocks (i.e. their permeability and porosity) stays the same. We conjecture that with decreasing *dx*, the *variance* of this distribution has to increase. As the blocks become smaller and smaller, some of them will likely represent a void of the porous matrix (thus becoming very permeable) and others will likely represent the bulk of the porous matrix (thus becoming negligibly permeable). Note that this idea goes directly against the continuum mechanics paradigm! To stress the contrast further, imagine we treated the problem of heat diffusion by the semi-continuum approach presented above. The heat conducting material would be cut up into blocks and heat would flow between the adjacent blocks according to Fourier’s Law. Each block would be described by the same heat conductivity coefficient. Next, the mesh would be refined. Naturally, the heat conductivity coefficients would not be affected by the refinement of the mesh. Taking the limit of the refinements (together with *dt* → 0) would yield the classical heat equation.

However, in the context of unsaturated porous media flow, the material coefficients cannot stay constant during the limiting process. As the blocks become smaller, the *variance* of the material parameters has to increase. If such a scaling is introduced, we believe a meaningful limiting process *dx* → 0 of the semi-continuum model can be performed. This cannot be done in one spatial dimension, since the increase in the variance of the characteristics would lead to “topological bottlenecks”, i.e. blocks with permeability low enough to jam the flow completely. In two or three spatial dimensions, these bottlenecks will also form but will be circumvented easily by the flow. Moreover, we believe that these bottlenecks will also produce the rich structure of spatial patterns of finger flow, which is observed in experiments but so far has not been reproduced by any model with continuous saturation. The MMIP model of Glass *et al*.^28^ has been so successful because it correctly captures the *variance* of the wetting and draining pressure across the material. However, since it still treats saturation as a discrete quantity, it cannot be used to reproduce any of the results we present here. Since this paper deals with one dimensional flow, the limiting process is not addressed here.

Unlike the dependence of the model on *dx*, its dependence on time step *dt* is similar to the behavior of explicit numerical schemes for parabolic partial differential equations. For *dt* small enough, the solution becomes stable and independent on further decrease in *dt*. The choice of *dt* depends mainly on the influx *q*_0_ and on the parameter *K*_*PS*_ which characterizes the scanning curves in the region between the main wetting and draining branches of the retention characteristics. The time step *dt* decreases with increasing *q*_0_ and *K*_*PS*_.

Averaging of hydraulic conductance has been used before^36,37^ and heavily criticized since then^13^. Nieber used the averaging process in a numerical solution to RE. That was proven wrong by Eliassi *et al*.^13^ who showed that his “numerical solution” was far from the true solution to RE. Thus, by the averaging process, Nieber obtained an incorrect numerical solution to RE. Today we now know that RE is inappropriate for finger flow modeling^35^. In the present semi-continuum model, the averaging process is not used to obtain a numerical solution to any differential equation. The averaging process is based on the studies of the so called equivalent hydraulic conductivities of spatially varying media^38^. There is theoretical justification to use the harmonic mean of conductivities (as a lower bound) in case of a medium stratified perpendicular to the flow direction^38^. This is similar to the situation at the fingertip where an almost saturated block is in contact with an almost dry block beneath. In a series of numerical experiments in a random pore networks, Jang *et al*.^38^ report results which are mostly consistent with the averaging process using the geometric mean. Thus, the geometric mean of relative permeability of the neighboring blocks is used in our model.

Experiments show that the behavior of the finger tip edge is crucial for the flow pattern. The overshoot can be understood as fluid piling up behind the fingertip because the dry medium ahead of the fingertip has insufficient hydraulic conductance thus the flux across the fingertip cannot keep up with the flux in the tail of the finger. The detailed mechanism of the fingertip advance matters here. The fluid menisci “jump” from grain to grain, rather than flow in a continuous way. That is why the overshoot behavior so much depends on the shape of the grains, as reported by DiCarlo^33^. Materials with identical macroscopic characteristics exhibit different overshoot behavior. It may be possible to model this process if we knew the exact positions and shapes of the grains at the fingertip. However, such detailed knowledge is neither available nor wise to incorporate in a porous medium flow model. Our model accounts for this “burst-and-hold” behavior by the following fast positive feedback: The block beneath the finger tip is dry, thus its conductivity is very low. The flux into the dry block is thus negligible and fluid piles up behind the fingertip. Once the pressure at the front increases enough and the previously dry block starts filling with water, the flux from the finger tip increases very quickly (as the third power of the saturation), making the block even more wet, which in turn increases the flux even more. This positive feedback fills the dry block with fluid almost instantly. This makes the finger “jump” ahead by one block. Notice that modeling the flux between the blocks by means of the geometric average is crucial in this process. It should also be stressed that it is crucial to take into account the hysteresis of the retention curve which prevents the oversaturated fingertip to drain into the undersaturated tail.

In view of this burst-and-hold behavior, it is interesting to recall the oscillations in pressure that the semi-continuum model exhibits. We do not know whether this behavior is an artifact of the discrete nature of the model or if it reflect reality. We note that the period of the oscillation is of the order of ten seconds in our model. It may be difficult to detect such fast changes by conventional tensiometers.

The microscopic origin of the “burst-and-hold” mechanism has been debated for a long time. Capillary pressure arises from the shapes of the water-air interfaces on the pores are dictated by the geometry of the pore space and the incompressibility of the flow. It is probable that velocity dependent effects also play a role^39,40^. Hoffman^41^ describes the dependence of the contact angle on the velocity of the flow. Steenhuis *et al*.^39^ use this result to predict the capillary pressure in the fingertip for various levels of influx and initial saturation. They assume that water at the fingertip advances by bursting through a single pore with a velocity high enough to cause a decrease in the contact angle. This causes the pressure to build up in the tip of the finger. There is experimental evidence in support of this mechanism^42^. Whatever the microscopic origin of the capillary pressure is, a semi-continuum model has to capture its effect in terms of quantities that are measurable on the level of the blocks. In our case, the macroscopic manifestation of these pore-scale processes is the low relative permeability between the wet block at the fingertip and the dry block directly beneath.

Let us stress again that the presented model is entirely based on well-known physical principles and material properties. The coefficient *K*_*PS*_ may be an exception–it is not usually assumed that all the scanning curves between the main wetting and draining branch of the retention curve are lines with a large gradient. However, any measurement is difficult here and sometimes an estimate of the gradient of the scanning curves is the only option.

**Figure 9:**
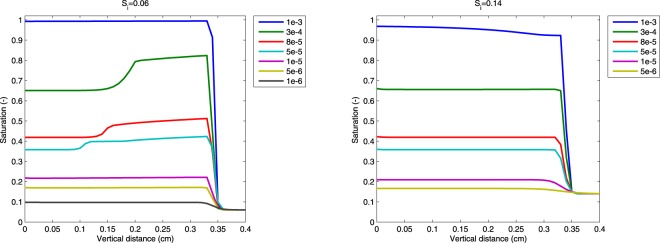
Smooth transition from overshoot behavior for large fluxes to no-overshoot behavior for small fluxes. Various influx magnitudes [m/s] color coded. Left panel: initial saturation *S*_*i*_ = 0.06. Right panel: initial saturation *S*_*i*_ = 0.14. No visible saturation overshoot is present for this initial saturation. However, the model shows a small but distinct pressure overshoot (see the Capillary pressure overshoot section).

Let us also note that the presented model is in no way “tuned” to capture saturation overshoot. When diffusion-like monotonic behavior is expected, the model captures it well, too (see Fig. 9 for a smooth transition from overshoot to no-overshoot behavior).

By an easy extension, the model can also be used to under fully or partially saturated conditions. If a block becomes fully saturated, the (negative) capillary pressure becomes zero and (positive) hydrostatic pressure appears. The hydrostatic pressure is computed using the height of the water column (i.e. the height of fully saturated blocks) above the considered block. Thus, in Eq. 7, hydrostatic pressure difference appears on the right hand side instead of the capillary pressure difference.

## Conclusion

A one dimensional semi-continuum model of steady infiltration into a homogeneous unsaturated porous medium is presented. The model fully rests on well-known theoretical and experimental concepts developed in soil physics^1^ and soil hydrology^43^. From mathematical point of view, the model is a cellular automaton based on the Macro Modified Invasion Percolation concept of dividing the porous medium into blocks which are not infinitesimal and are assumed to retain the characteristics of a porous medium. In discrete time steps (which are considered infinitesimal), the cellular automaton repeats three successive rules: (1) saturation update in each block based on known fluxes between the neighboring blocks, (2) pressure update in each block based on saturation known from the previous step and the retention characteristics of the material, (3) flux update between neighboring blocks based on the Darcy-Buckingham Law and a geometric mean of the hydraulic conductivity of the two blocks.

The model captures well all the features of one dimensional unsaturated porous media flow (i.e. three dimensional flow in a thin tube), especially finger flow, including the saturation overshoot, capillary pressure overshoot and their dependence on initial saturation of the medium and influx intensity. The shape of the saturation overshoot profile and its evolution in time and space also agrees with observations.

The limit of the proposed model for the block size going to zero is a subtle issue and cannot be considered in one spatial dimension for topological reasons. In performing the limit, the variability of the characteristics of the blocks (permeability, porosity, and retention characteristics) has to be considered carefully. Extension of the model to two and three spatial dimensions will be considered next, together with the limit for block size going to zero.

We believe that the semi-continuum approach has the potential to bridge the gap between discrete models of unsaturated porous media flow and continuum models.

## Data Availability

The code of the semi-continuum model written either in MatLab or Fortran is available from the corresponding author on reasonable request.
